# Eradication of *Mycoplasma pneumoniae* biofilm towers by treatment with hydrogen peroxide or antibiotic combinations acting synergistically

**DOI:** 10.1371/journal.pone.0329571

**Published:** 2025-08-28

**Authors:** Rasha A. Fahim, Zoë E. D. Rodriguez, Zachery Oestreicher, Nathan R. Schwab, Natalie E. Young, Kavita Shrestha, Mitchell F. Balish

**Affiliations:** 1 Department of Microbiology, Miami University, Oxford, Ohio, United States of America; 2 Center for Advanced Microscopy and Imaging, Miami University, Oxford, Ohio, United States of America; University of Buea, CAMEROON

## Abstract

*Mycoplasma pneumoniae* is an important chronic, asthma-associated pathogen that is increasingly antibiotic-resistant. These bacteria have highly reduced genomes and lack a cell wall and numerous other antibiotic targets. They form biofilm towers after prolonged growth both axenically and on tissue culture cells. The biofilm towers have features associated with chronic infection: they are highly resistant to erythromycin and have substantially increased resistance to complement, although they are sensitive to a combination of the two. This work sought to characterize the profile of agents that could eradicate *M. pneumoniae* biofilm towers. Biofilm towers were found to provide no defense against H_2_O_2_, an *M. pneumoniae* virulence factor whose production is severely attenuated during biofilm tower growth. Checkerboard assays revealed that dual combinations of erythromycin, moxifloxacin, and doxycycline acted synergistically against two strains of *M. pneumoniae*. Crystal violet assays suggested that pairs of these agents, when used at clinically relevant concentrations, had substantial efficacy against pre-formed biofilm towers, but scanning electron microscopy revealed that the eradication of biofilm towers was even more complete than crystal violet assays indicated. Although the use of fluoroquinolones and tetracyclines in children, who are the most frequently infected population, is not preferred over macrolides due to potential side effects, this work shows that synergistic interactions among therapeutic agents provide potential clinical paths to substantially reducing or eradicating *M. pneumoniae* biofilms, thereby decreasing morbidity. Furthermore, the sensitivity to H_2_O_2_ suggests that small-molecule therapeutics may also be suitable for biofilm clearance.

## Introduction

*Mycoplasma pneumoniae* is a common community-acquired pathogen of the human upper respiratory tract, spread by droplet inhalation. This bacterium, underdiagnosed because of its fastidious growth requirements, is most frequently the causative agent of a spectrum of disease symptoms that includes tracheobronchitis and pneumonia, especially but not exclusively in school-age children [1]. Epidemics typically occur every 1–5 years; a gap during and beyond the COVID-19 pandemic occurred, but the organism has returned, causing widespread hospitalization of children [1–3]. Although it is rarely lethal, *M. pneumoniae* is frequently associated with a large range of extrapulmonary conditions, including encephalitis, endocarditis, and Stevens-Johnson syndrome [1]. Even though immunocompetent individuals can clear respiratory infections, and treatment with antibiotics to which the organism is susceptible can be very successful in dispelling symptoms, infections tend to be long-term in nature. Indeed, *M. pneumoniae* is often detectable long after abatement of symptoms [1]. Moreover, *M. pneumoniae* is associated with asthma and/or an asthma-like condition [1,4]. Infections appear to predispose children to asthma, and *M. pneumoniae* is detectable in patients with an asthma-like hyperreactive airway condition that, unlike true asthma, is ameliorated by antibiotic treatment.

Despite considerable efforts, there is no successful vaccine against *M. pneumoniae*, and treatment is restricted to a limited range of antibiotics [1]. Mycoplasmas are genome-reduced parasites that lack cell walls and outer membranes and have an extremely limited set of anabolic pathways, all of which are important targets of broad-spectrum antibiotics like beta-lactams, polymyxins, and sulfonamides. Macrolides are the first choice for treatment for upper respiratory infections and have historically been highly successful at eliminating symptoms in patients infected with *M. pneumoniae*. Resistance to antibiotics, however, is a well-established and growing problem, resulting in complications in treatment of patients and adverse outcomes [5]. Indeed, over the past quarter-century, macrolide-resistant *M. pneumoniae* strains have been isolated from patients with increasing frequency, with about 15% of clinical isolates from the United States exhibiting this property, and over 75% in east Asia [6]. Fluoroquinolones and tetracyclines, while effective against *M. pneumoniae*, have historically been associated with detrimental side effects from long-term use in children, limiting their clinical application. However, recent work suggests that these drugs can be efficacious against *M. pneumoniae* and other bacterial pathogens in pediatric patients with minimal toxicity [7].

Despite being an obligate parasite of humans *in vivo*, *M. pneumoniae* can be cultured axenically in a laboratory setting. It is typically grown in any of various undefined media, though limited growth is possible in some minimized and semi-defined formulations [8,9]. Wild-type *M. pneumoniae* cells grow attached to the surface of a flask, where they can propel themselves by gliding motility. Motility is not associated with chemotaxis but is essential in some way for avoiding clearance and causing disease [10]. Whether grown axenically or on BEAS-2B respiratory tissue culture cells, *M. pneumoniae* forms distinct mound- or dome-shaped biofilm towers over a period of several days, and this growth is independent of motility [11,12].

Importantly, these towers, which grow from aggregates of bacteria [12], have features that are consistent with the ability to cause chronic disease. As biofilm towers grow, the bacteria become increasingly resistant to the macrolide erythromycin (ERY), reaching a point at which it is ineffective at 512 µg ml^-1^, 8,500−128,000 times the minimal inhibitory concentration (MIC) under normal assay conditions in the absence of biofilm towers [12,13]. In addition, biofilm towers show a 3- to 10-fold increase in resistance to complement as they grow [12,13]. Biofilm towers are also marked by a decline in production of several virulence factors, including H_2_O_2_, H_2_S, and the ADP-ribosylating and vacuolating CARDS toxin [13]. This attenuation of virulence factors is consistent with taking up long-term residence and limiting excessive host damage and immune response. Indeed, given that host cells also produce H_2_O_2_ to defend against bacteria, the use of this molecule by *M. pneumoniae* as a virulence factor raises interesting questions about the conditions under which *M. pneumoniae* might produce it. Taken together, these data suggest that the biofilm tower state is a distinct developmental phase of *M. pneumoniae* with properties that could cause the long-term disease conditions with which this organism is associated.

It is therefore important to learn what treatments could eliminate *M. pneumoniae* biofilm towers, neutralizing this mode of self-protection. Although a combination of ERY and complement is considerably effective in killing *M. pneumoniae* towers [12], complement is not likely to be a physiologically relevant mechanism of attack in the respiratory tract, and the concentration of ERY used in these experiments was beyond the physiological range. This work sought to test whether biofilm towers might be sensitive to antimicrobial agents aside from ERY, including the clinically important but less preferred antibiotics doxycycline (DOX) and moxifloxacin (MOX), as well as H_2_O_2_ as a model for small molecules. This work indicates that *M. pneumoniae* biofilm towers are not at all protected from H_2_O_2_, and that pairs of antibiotics have profound synergistic effects that virtually eliminate biofilm towers *in vitro*. Although it is not clear what specific combinations of molecules might be the most clinically appropriate, this work demonstrates a path toward therapeutic strategies to potentially reduce the incidence, length, and severity of chronic disease.

## Materials and methods

### Bacterial strains and growth conditions

*Mycoplasma pneumoniae* wild-type strains M129 and 19294 (gift of T.P. Atkinson, University of Alabama at Birmingham), representing each of the two subtypes of the species, were chosen for use so that the genetic diversity of *M. pneumoniae* is well-represented. They were inoculated into tissue culture flasks containing 10 ml of SP-4 broth [14] and incubated at 37°C until the color changed to yellow (mid-to-late log phase), and frozen stocks were made by scraping the cells into the broth, dividing it into 1-mL aliquots, and storing at −80°C for stocks. Stocks were used to inoculate SP-4 broth in 24- or 96-well plates. Incubation of *M. pneumoniae* was carried out at 37°C for all experiments.

### Antimicrobial agents

ERY, DOX, and MOX were obtained from Sigma Aldrich, Alfa Aesar, and Thermo Fisher Scientific Chemicals, respectively. The ERY stock was at 25.6 mg ml^-1^ in ethanol; the MOX stock was at 2.048 mg ml^-1^ in ultra-pure water, and the DOX stock was at 20 mg ml^-1^ in ultra-pure water. All stocks were filter-sterilized and stored at −20°C. All dilutions were made using SP-4 broth. 30% H_2_O_2_ (Thermo Fisher Scientific) was stored at room temperature and dilutions were made in SP-4 broth.

### Minimum inhibitory concentration (MIC) testing

MIC assays were conducted as previously described [13,15]. Briefly, stocks were syringed through a 26-g needle multiple times, followed by dilution in SP-4 broth to achieve a final inoculum of 1.0 X 10^4^ CFU ml^-1^ for antibiotic testing and 1.14 X 10^4^ CFU ml^-1^ for H_2_O_2_ assays, in accordance with guidelines for mycoplasma testing [14]. To evaluate the MICs of antibiotics on non-biofilm tower *M. pneumoniae*, bacteria were incubated in tubes for two hours at 37°C before inoculation into 96-well plates containing SP-4 with antibiotics. For H_2_O_2_ MIC measurement, H_2_O_2_ was added at the time of inoculation. Each compound was freshly prepared and diluted in a series of ten twofold dilutions starting at 1 μg ml^-1^ for antibiotics, or at 2% H_2_O_2_. The plates were incubated until the growth control changed color from red to yellow. The MIC was the lowest concentration of antimicrobial for which there was no color change. For antibiotic experiments, the starting and final concentrations of each antibiotic were used as drug controls, SP-4 medium was used as a sterility control, ethanol was used as a vehicle control for ERY, and SP-4 with the inoculated bacteria and no added antibiotics was used as a growth control. For H_2_O_2_ assays, SP-4 with the highest concentration of H_2_O_2_ was used as a sterility control, and SP-4 without H_2_O_2_ was used as a growth control. Every experiment was performed in triplicate.

### *In vitro* synergy testing

The interactions between different antibiotics with respect to *M. pneumoniae* were tested using the checkerboard broth microdilution assay as previously described [16,17]. Briefly, the antibiotics were diluted to start with twice the MIC of each antibiotic. Then, decreasing concentrations of both antibiotics were added to each well of a 96-well plate, except for the last row and column, in which only one of either antibiotic was added. The last well was left as a growth control without any antibiotics added. Then, *M. pneumoniae* was inoculated in all wells and incubated until the growth control changed color. The interactions between antibiotic combinations were assessed by determining the Fractional Inhibitory Concentration Index (FICI) was calculated as (MIC of drug A in combination/ MIC of drug A alone) + (MIC of drug B in combination/ MIC of drug B alone) [16,17]. Synergy was defined as an FICI of <0.5, additive effect was defined as an FICI of 0.5 to 4, and antagonism was defined as an FICI of >4.0. Experiments were performed in triplicate.

### Susceptibility of preformed biofilms to antibiotics and H_2_O_2_

*M. pneumoniae* was cultured in SP-4 broth in 24-well plates for 48−72 hours to form biofilm towers, and then the medium was removed and replaced with medium containing antibiotics or H_2_O_2_ and grown another 48 hours. Antibiotic concentrations started at 512 μg ml^-1^; H_2_O_2_ concentrations started at 0.032%. For evaluation of biofilm density by crystal violet (CV) assay, spectrophotometric analysis was carried out immediately after the second 48 hours as previously described [11]. Briefly, wells were stained with 0.1% CV for 10 minutes, washed 3X in phosphate-buffered saline, and air-dried for 10 minutes. After destaining with 95% ethanol for 10 minutes, the OD_600_ was measured on a spectrophotometer. OD_600_ values were directly compared across samples. For viability experiments, the medium was removed again, fresh medium was added, and cultures were incubated for another 24 hours to test whether there was a color change from red to yellow showing continued viability. Experiments were performed in triplicate.

### Pretreatment of media with H_2_O_2_

SP-4 broth was treated for 48 hours at 37°C with 0.008% H_2_O_2_ and then neutralized for 1 hour with 10 U of catalase (Millipore Sigma). *M. pneumoniae* was inoculated into 24-well plates with this H_2_O_2_-treated medium and grown as described above until untreated SP-4 in a control well turned yellow. Other controls included medium treated with only H_2_O_2_ or only catalase. Experiments were performed in sextuplicate.

### Comparison of crystal violet (CV) and scanning electron microscopy (SEM)

Biofilms were grown as described above, and after 48 hours, the medium was replaced with pairs of antibiotics at 3 μg ml^-1^ for ERY and MOX and 4 μg ml^-1^ for DOX. After another 48 hours, CV assays were carried out as described above. For SEM analysis, the biofilms were fixed on the glass coverslips as previously described [18] and the samples underwent critical point drying (Tousimis, Samdri 780A) and sputter-coated with approximately 20 nm of gold (Denton, Desktop II), following the previously described method [19]. Samples were visualized using a Carl Zeiss Supra 35 VP field emission SEM operating at 5 keV at the Miami University Center for Advanced Microscopy and Imaging. Experiments were performed in triplicate.

### SEM of H_2_O_2_-treated *M. pneumoniae*

*M. pneumoniae* was grown in SP-4 broth as described above for 3 or 6 days with or without H_2_O_2_. Samples were treated as described above and in the Fig 2 legend. SEM was carried out as described above.

### Statistical analysis

Treatments were analyzed with one-way ANOVA and post-hoc Tukey HSD, considering differences with *p* < 0.01 to be statistically significant.

## Results

### Sensitivity of *M. pneumoniae* biofilm towers to H_2_O_2_

**Fig 1:**
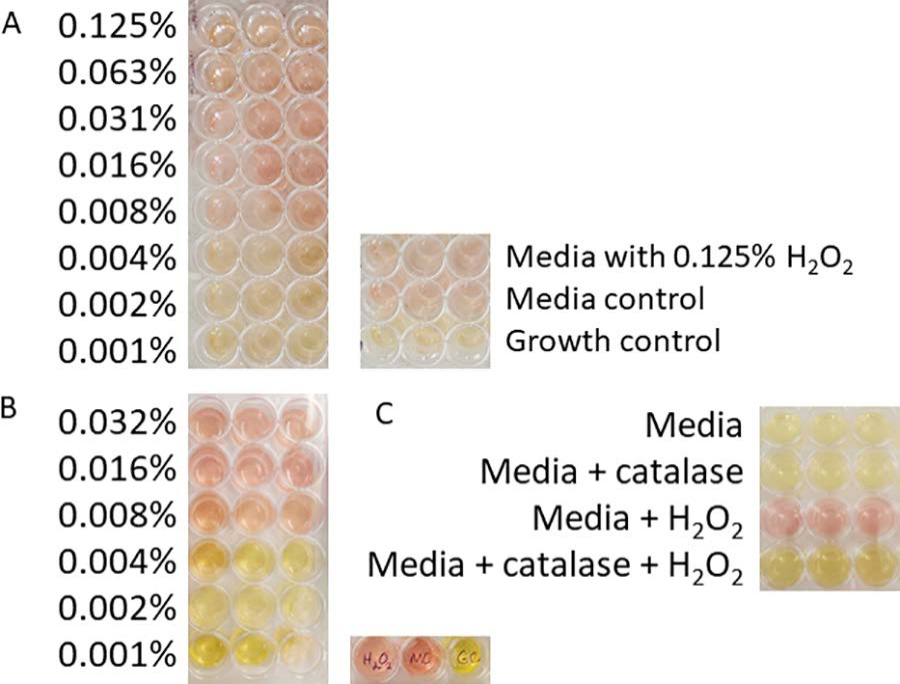
Sensitivity of *M. pneumoniae* strain M129 to H_2_O_2_. Red color of SP-4 broth indicates no growth; yellow indicates growth. (A) MIC of H_2_O_2_ for *M. pneumoniae*. H_2_O_2_ was included during inoculation. For the left panel, concentrations of H_2_O_2_ are indicated to the left, and the three columns of wells are triplicates. For the right panel, control conditions are indicated to the right. (B) MIC of H_2_O_2_ for pre-formed *M. pneumoniae* biofilm towers. H_2_O_2_ was added after 3 days post-inoculation, after towers had formed. For the left panel, concentrations of H_2_O_2_ are indicated to the left, and the three columns of wells are triplicates. For the right panel, the well labeled “H_2_O_2_” had SP-4 broth with 0.032% H_2_O_2_ and was not inoculated with *M. pneumoniae*; the well labeled “MC” had SP-broth with no H_2_O_2_ and was not inoculated with *M. pneumoniae*; the well labeled “GC” received no H_2_O_2_ after inoculation and grew 6 days. (C) Growth of *M. pneumoniae* in SP-4 broth pretreated with 0.008% H_2_O_2_ and/or 10 U catalase, as indicated on the left. The three columns of wells are triplicates.

**Fig 2:**
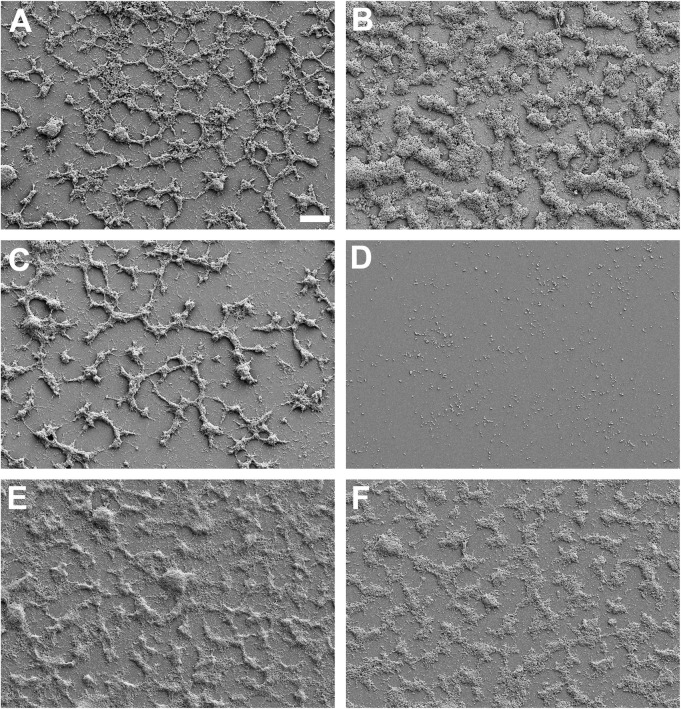
Scanning electron micrographs of *M. pneumoniae* strain M129 biofilm towers showing the effects of H_2_O_2_ treatment for 3 or 6 days. *M. pneumoniae* grown for: (A) 3 days without H_2_O_2_; (B) 6 days without H_2_O_2_ (C) 3 days with 0.004% H_2_O_2_; (D) 6 days with 0.004% H_2_O_2_; (E) 3 days without H_2_O_2_ and then 3 days with 0.004% H_2_O_2_; (F) 3 days without H_2_O_2_ and then 3 days with 0.008% H_2_O_2_. Scale bar, 10 μm.

*M. pneumoniae* biofilm towers, which begin to dominate *in vitro* cultures at 48 hours post inoculation [11], are characterized by complete resistance to ERY and recalcitrance to complement [11]. Significantly, in a submerged tissue culture model a combination of the two eliminates all but a few percent of the bacteria [12]. The ability of *M. pneumoniae* strain M129 biofilm towers to resist H_2_O_2_, a cytotoxic molecule that *M. pneumoniae* produces [20,21] but whose production is sharply reduced coincident with biofilm tower formation *in vitro* [13], was tested. The MIC of H_2_O_2_ was found to be 0.008% both when H_2_O_2_ was included during inoculation (Fig 1A) and when it was added to media after biofilms had formed (Fig 1B). Therefore, biofilm towers offer no protection against this molecule, in sharp contrast to ERY [13]. It was possible that the effect of H_2_O_2_ was indirect, such as by damaging essential components of the medium. To test this possibility, the medium was pre-treated with 0.008% H_2_O_2_, the MIC, for 48 hours, which is expected to be long enough to oxidize any medium components. Then the medium was incubated with catalase to remove the H_2_O_2_ before inoculation with *M. pneumoniae*. This pretreatment resulted in the same MIC, indicating that oxidation of medium components by H_2_O_2_ was not responsible for failure of *M. pneumoniae* to grow (Fig 1C). Interestingly, with 0.004% H_2_O_2_, which is below the MIC, biofilm towers were robust after 72 hours of growth as visualized by SEM, but at 144 hours the surface was cleared of biofilm towers, suggesting long-term weakening of the towers by H_2_O_2_ even below the formal MIC (Fig 2A-D). Incubation of preformed towers for 72 hours in medium containing 0.008% H_2_O_2_ arrested their growth (Fig 2E-F).

### Resistance of *M. pneumoniae* biofilm towers to antibiotics of clinical importance

**Fig 3:**
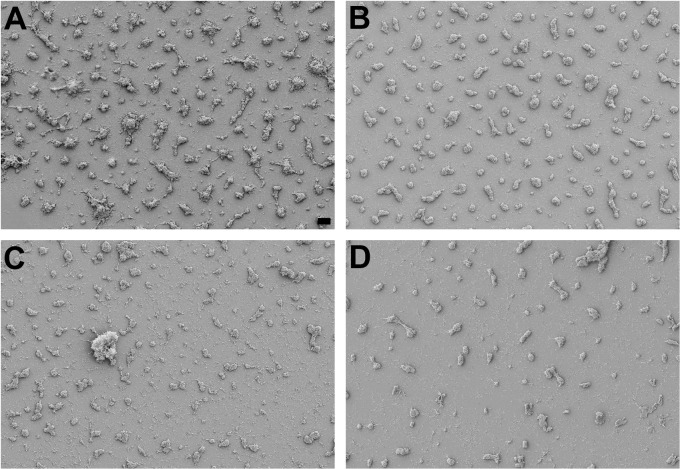
Scanning electron micrographs of *M. pneumoniae* strain M129 biofilm towers treated with individual antibiotics showing minimal effect on the towers. (A) Control, *M.* grown for 96 hours without antibiotics. (B-D) Grown for 48 hours before addition of antibiotics and incubated another 48 hours before imaging: (B) ERY, 3 μg ml^-1^; (C) MOX, 3 μg ml^-1^; (D) DOX 4 μg ml^-1^. Scale bar, 10 μm.

To test whether complete resistance of *M. pneumoniae* biofilm towers was a phenomenon specific to ERY, a large (>800-Da) molecule which might have limited penetrance, two smaller (~400-Da) and medically relevant antimicrobials, MOX and DOX, were tested on *M. pneumoniae* biofilms grown for 48 hours. *M. pneumoniae* is sensitive to these antibiotics in standard MIC assays [22]. However, when introduced at 3–4 μg ml^-1^, concentrations that are achievable in plasma [23–25], these drugs were ineffective in eliminating preformed biofilm towers of *M. pneumoniae* strains M129 (Fig 3) and 19294 (not shown), representing diverse genetic backgrounds within the species. CV staining showed a modest reduction of no more than two-fold (Table 1; S1 Data), confirming the relative ineffectiveness of these agents against pre-formed biofilm towers.

**Table 1:** Reduction of preformed *M. pneumoniae* biofilm towers after treatment with antibiotics for 48 hours.

Treatment type	Antibiotic	Percent CV stain remaining (± SD) Strain M129	Percent CV stain remaining (± SD) Strain 19294
Monotherapy	ERY^a^	59.6 ± 4.3	55.5 ± 9.0
	DOX^b^	64.1 ± 8.5	55.2 ± 6.9
	MOX^a^	49.7 ± 8.9	44.2 ± 11.7
Dual therapy	ERY and DOX	16.7 ± 4.5	22.4 ± 5.7
	ERY and MOX	13.9 ± 5.2	11.1 ± 1.6
	DOX and MOX	12.6 ± 2.7	20.3 ± 1.6

^a^ERY and MOX, 3 µg ml^-1^;

^b^DOX, 4 µg ml^-1^.

### Synergy of antibiotic pairs against *M. pneumoniae* biofilm towers and non-tower bacteria

Because the individual antibiotics were not effective against *M. pneumoniae* biofilm towers, pairs of these antibiotics were tested. First, the efficacy of pairs of antibiotics against pre-biofilm tower *M. pneumoniae* cultures was evaluated using checkerboard assays. When incubated with cultures before formation of biofilm towers, although there were some strain-dependent differences, all three combinations of two drugs had FICI values that ranged from 0.14 to 0.50, indicating either borderline or full-fledged synergy (Table 2; S2 Data).

**Table 2:** Combinatorial effects of antibiotics against *M. pneumoniae* prior to biofilm tower formation.

Antibiotic combination	FICI for strain M129	Action on strain M129	FICI for strain 19294	Action on strain 19294
ERY and DOX	0.37	synergistic	0.50	additive/synergistic
ERY and MOX	0.50	additive/synergistic	0.37	synergistic
DOX and MOX	0.14	synergistic	0.50	additive/synergistic

**Fig 4:**
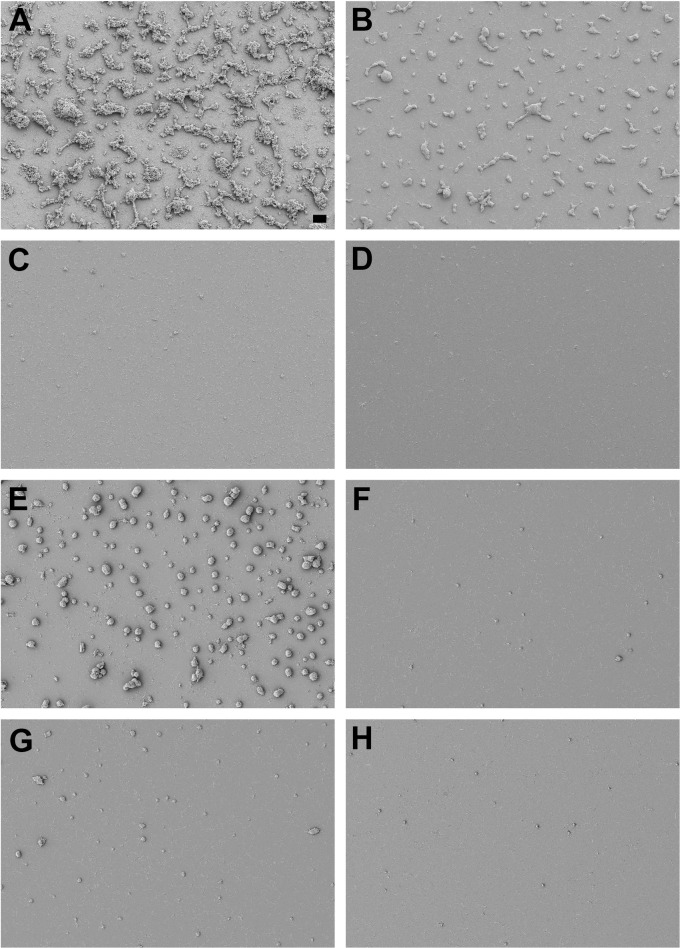
Scanning electron micrographs of *M. pneumoniae* biofilm towers treated with pairs of antibiotics showing profound effects on the towers. Strains M129 (A-D) and 19294 (E-H) were grown for 48 hours before addition of different combinations of antibiotics. Dose concentrations: ERY and MOX, 3 μg ml^-1^; DOX 4 μg ml^-1^. Cultures were incubated another 48 hours before imaging. (A, E): no antibiotic (96 hours of growth); (B, F): ERY and DOX; (C, G): ERY and MOX; (D, H): DOX and MOX. Scale bar, 10 μm.

In contrast to individual antibiotics, pairs of antibiotics profoundly reduced or eliminated preformed *M. pneumoniae* biofilm towers, although there was a moderate disparity between results from CV assays and SEM visualization. By CV assay, each pair of antibiotics at 3–4 μg ml^-1^ showed a marked reduction, leaving only 11.0–22.4% of stain (Table 1). For both strains M129 and 19294, treatment with ERY + DOX resulted in ~80% eradication by CV (Table 1). By SEM, for strain M129, treatment with ERY + DOX resulted in smaller towers as compared with a 96-hour untreated control (Fig 4A),and appeared to reduce nearly all *M. pneumoniae* cells residing between the towers to debris and fragments (Fig 4B). However, for strain 19294 (Fig 4F), towers were mostly eliminated compared to the control (Fig 4E), leaving behind a lawn of individual bacterial cells. Although it is impractical to quantify the total volume of cells by SEM, there appeared to be far less volume of strain 19294 remaining than of strain M129, which does not agree with the very similar CV results (Table 1). For ERY + MOX, CV results indicated elimination of nearly 90% of either strain of *M. pneumoniae* (Table 1). By SEM, for both strains, only very small towers were scattered among the intact individual cells or debris (Fig 4C, 4G). For MOX + DOX, CV assays indicated 80–87% eradication (Table 1). However, for strain M129, SEM revealed almost complete eradication of all material (Fig 4D), and for strain 19294 there was some debris (Fig 4H).

## Discussion

Although *M. pneumoniae* is a structurally atypical bacterium, its ability to form antibiotic-resistant biofilm structures [13] is shared with many other bacteria. Nonetheless, there are some features of its lifestyle that tend to confound simple comparison with walled bacteria. For one, it is unclear whether *M. pneumoniae*, which can undergo gliding motility on surfaces but lacks flagella or any other means of propelling itself through liquid [10], has a truly planktonic phase. We have proposed that its primary means of spread through the respiratory tract and between individuals is through bacterial cells settling onto host cell surfaces, with bacterial aggregates then growing into biofilm towers [12]. In this model, it is not the deliberate escape from biofilm towers into the host extracellular space that is responsible for spread, but rather host-driven mechanical disruption of bacterial structures that releases bacterial cells into the flow of the mucociliary escalator. At this point they may either settle in a new location or be swept out.

It seems, then, that although these cells that are transiently disengaged from the substrate are present, the primary distinction among *M. pneumoniae* cell types during its life cycle is between adherent cells that are in biofilm towers and adherent cells that are not. The distinction between tower and non-tower cells is almost certainly the basis for differential susceptibility of *M. pneumoniae* to ERY, DOX, and MOX at different times during growth (Table 1). When the culture is dominated by adherent bacteria that are not in biofilm towers, it is sensitive to these drugs, but later, the culture is dominated by tower cells that resist them.

In addition to the previous observation that ERY, at a therapeutically unachievable concentration, together with complement can reduce biofilm amounts by over 90% [12], this work has identified two types of treatments that also go a long way toward eliminating biofilm towers. One is H_2_O_2_, a small, toxic, membrane-permeant molecule produced by both host cells and *M. pneumoniae* [21]. The susceptibility of biofilm towers to this molecule, as indicated by both MIC assays and SEM, is equivalent to that of non-tower cells; indeed, prolonged exposure even at lower concentrations can destroy towers (Figs 1, 2). It is therefore unlikely to be a coincidence that H_2_O_2_ production by *M. pneumoniae* is sharply attenuated at the time biofilm towers begin to form.

The degree to which *M. pneumoniae* biofilms appeared to be eradicated differed depending upon the type of analysis, which may be attributed to the close interaction between *M. pneumoniae* cells – even those not in biofilm towers – and surfaces. Although the CV assay is quantitative and the SEM images are not, the CV data generally suggest substantially greater resistance of the biofilms to antibiotics than the SEM images (Table 2; Fig 4). We propose that this discrepancy is at least partly attributable to the continued adherence of fragments of non-tower cells to the surface following treatment, which are anticipated to be capable of retaining CV but do not represent living organisms. This material is clearly visible all over most images (Fig 4). In addition, the very rich, undefined SP-4 medium appears to deposit a substantial and quite variable amount of material on the substrate (data not shown), tending to distort the background value for CV absorption. Therefore, for *M. pneumoniae*, the effectiveness of biofilm eradication is quite difficult to accurately assess quantitatively. The visual information from SEM images, however, is rather clear about either arrest or removal of biofilm towers. Despite the CV assay results, it appeared that pairs of antibiotics were very effective in eliminating *M. pneumoniae* biofilm towers.

Although individual antibiotics were ineffective (Fig 3), treatment with pairs of antibiotics of different classes were highly effective at eradicating biofilm towers, and they did so synergistically (Table 2; Fig 4). It is not entirely clear why two antibiotics, often at lower total concentrations than individual ones in a single-antibiotic treatment, would behave synergistically if physical penetration of the biofilm is the limiting factor. Presumably, physical damage by a single antibiotic would allow penetration of the same antibiotic, but the survival of some cells in biofilm towers treated with even the highest levels of single antibiotics (not shown) appears to refute that mechanism. The basis for this synergy is therefore more likely to include a biochemical component, with the arrest of two processes resulting in the strong damage to towers. However, this was also true for the combination of DOX and ERY, both of which target the translation apparatus. Although little evidence is present in the literature concerning synergy between tetracyclines and macrolides, possibly the simultaneous inactivation of the 30S and 50S subunits by these drugs is significant for an atypical organism like *M. pneumoniae*.

The sharp reduction in *M. pneumoniae* biofilm towers upon treatment with pairs of antibiotics at clinically relevant concentrations may be of therapeutic value. Moreover, the result that H_2_O_2_ is as toxic to *M. pneumoniae* cells in biofilm towers as it is to cells that are not (Figs 1, 2) suggests that small molecule therapeutics may be useful for biofilm elimination, although it is possible that the membrane permeability of H_2_O_2_ is at least equally important. The relative merits of the particular antibiotics tested in the present study are debatable, with fluoroquinolones and tetracyclines presenting various problems for children, albeit possibly with tolerable levels of risk [26]. A combination of DOX and the macrolide azithromycin was highly effective in curing patients infected with the closely related organism *Mycoplasma genitalium* [27], demonstrating the clinical potential for using combination therapy against mycoplasmas.

## Conclusion

This work shows that *M. pneumoniae* biofilms can be cleared, or at least reduced to levels that are expected to be manageable for most patients. This reduction or clearing can be achieved with combinations of clinically relevant antibiotics at relatively low concentrations, offering hope for prevention or amelioration of chronic infection. *M. pneumoniae* biofilm towers are also sensitive to prolonged exposure to low concentrations of H_2_O_2_, raising the possibility that therapeutically feasible small-molecule compounds could be used in chronically infected patients. Moreover, visual methods like SEM are superior to traditional CV staining for evaluating biofilm disruption for organisms like *M. pneumoniae*, which has a cell envelope with properties that differ from most bacteria.

## Supporting information

S1 Data Biofilm mass after antibiotic treatment. (XLSX)

S2 Data FICI data. (DOCX)
