# Supplementary material for: Eradication of Mycoplasma pneumoniae biofilm towers by treatment with hydrogen peroxide or antibiotic combinations acting synergistically
Source: PLoS One. 2025 Aug 28;20(8):e0329571. doi: 10.1371/journal.pone.0329571 (PMC12393767; doi:10.1371/journal.pone.0329571)
Supplement: S2 Data — (DOCX) [file pone.0329571.s002.docx]

FICI = (MIC of drug A in combination / MIC of drug A alone) + (MIC of drug B in combination / MIC of drug B alone)

**M129 FICI**

**MOX + DOX:**

MIC for MOX alone: 0.125 μg ml^-1^

MIC for DOX alone: 0.125 μg ml^-1^ 0.14

MIC for MOX in combination: 0.002 μg ml^-1^

MIC for DOX in combination: 0.0156 μg ml^-1^

**MOX + ERY:**

MIC for MOX alone: 0.125 μg ml^-1^

MIC for ERY alone: 0.008 μg ml^-1^ 0.5

MIC for MOX in combination: 0.03125 μg ml^-1^

MIC for ERY in combination: 0.002 μg ml^-1^

**ERY + DOX:**

MIC for ERY alone: 0.008 μg ml^-1^

MIC for DOX alone: 0.125 μg ml^-1^ 0.37

MIC for ERY in combination: 0.002 μg ml^-1^

MIC for DOX in combination: 0.0156 μg ml^-1^

**19294**

**MOX + DOX:**

MIC for MOX alone: 0.125 μg ml^-1^

MIC for DOX alone: 0.125 μg ml^-1^ 0.5

MIC for MOX in combination: 0.03125 μg ml^-1^

MIC for DOX in combination: 0.03125 μg ml^-1^

**MOX + ERY:**

MIC for MOX alone: 0.125 μg ml^-1^

MIC for ERY alone: 0.008 μg ml^-1^ 0.37

MIC for MOX in combination: 0.0156 μg ml^-1^

MIC for ERY in combination: 0.002 μg ml^-1^

**ERY + DOX:**

MIC for ERY alone: 0.008 μg ml^-1^

MIC for DOX alone: 0.125 μg ml^-1^ 0.5

MIC for ERY in combination: 0.002 μg ml^-1^

MIC for DOX in combination: 0.03 μg ml^-1^
